# Changes in Stress Following Wage Increases for Early Childhood Educators

**DOI:** 10.1007/s10643-024-01666-0

**Published:** 2024-04-17

**Authors:** Randi A. Bates, Jaclyn M. Dynia

**Affiliations:** 1 College of Nursing, University of Cincinnati, 310 Proctor Hall 3110 Vine St, Cincinnati, Ohio 45221 USA; 2 SproutFive, Columbus, Ohio USA

**Keywords:** Educators, Stress, Depression, Anxiety, Wages, Salaries

## Abstract

Early childhood educators have one of the most important roles in society, helping the youngest generation flourish, yet educators earn some of the lowest wages. Given their disparate financial value, educators have unsurprisingly reported high stress. Educators’ high stress and low wages may affect their health, workplace turnover, and children’s development. Here, we observed whether natural wage increases were associated with reduced stress in educators during the 2021–2022 academic year. Across two time points (TPs), 67 center-based educators of children 0–5 years old completed surveys on their wages and psychological stress (perceived stress, depression, and anxiety). Fifty educators provided hair cortisol concentration to represent chronic physiological stress. Over about three months, educators received a mean wage increase of $1.85/hour. Before the wage increase, minoritized educators earned about $1.50 less than White educators, but earnings were more similar at the second TP. Educators’ depression at the start of the academic year was related to their next three months’ of physiological stress. Over time, educators’ stress remained high and was unassociated with wage changes. Our results suggest a concentrated examination into eliminating wage inequities and reducing the stress of educators.

**Supplementary Information:**

The online version contains supplementary material available at 10.1007/s10643-024-01666-0.

Early childhood educators (hereafter “educators”) may be experiencing critically severe levels of stress, defined as excessively high or prolonged activation of stress response systems that increase one’s risk for adverse health (Shonkoff et al., 2012). For example, recently published work through the COVID-19 pandemic shows that about 50% of educators experienced depression (Elharake et al., 2022) and moderate to severe perceived stress (Swigonski et al., 2021). In comparison, available national estimates during the pandemic showed that about 30% of adults experienced depression (Ettman et al., 2020, 2022; Rosenberg et al., 2021). While mental health is critical to educators’ well-being, it may also negatively affect their long-term health (Anda et al., 2006; Cohen et al., 2007), the well-being of their families (Justice et al., 2019; Masarik & Conger, 2017), and the young children in their classrooms (Caven et al., 2021; Kwon et al., 2021; Silver & Zinsser, 2020; Smith & Lawrence, 2019; Whitaker et al., 2015). Thus, there is a critical need to identify targets to reduce educators’ stress.

One important target to reduce educators’ stress may be their wages. Recent work and theory suspect low wages are a major culprit of educators’ stress (Kwon et al., 2021a; Otten et al., 2019). Despite their challenging workload, educators’ wages are less than 98% of other occupations (McLean et al., 2021). Educators’ median hourly wage of $13.22/hour (U.S. Bureau of Labor Statistics, 2021) is an unlivable wage for a single adult without children across most of the United States (McLean et al., 2021). These low wages were the strongest predictor of educator turnover (Caven et al., 2021) and may have resulted in many educators relying on public assistance (U.S. Department of the Treasury, 2021). Because educators are disproportionately female and of color (McLean et al., 2021; Paschall et al., 2021), educators’ low wages may also contribute to racial and gender pay-related disparities in the United States. Hence, educators’ disproportionately low wages may contribute to their stress due to relative and absolute poverty, which may also contribute to their social exclusion as they do not have an equal opportunity to financially survive.

However, increasing educators’ wages in the United States has been difficult. One of the reasons for the difficulty is, unlike the financial support for its Kindergarten-12th grade system, the United States provides insufficient subsidized financial support for its early childhood education and care structure (Grunewald & Davies, 2011; Johnston, 2023, April 8). Indeed, the lack of financial support for early childhood education and care in the United States is comparably abysmal to that of many developed countries. For example, out of the 35 Organization for Economic Cooperation and Development (OECD) member countries, the United States has the fifth highest income inequality and spends the seventh least on public early childhood education and care (Organization for Economic Cooperation and Development, 2023a, 2023b). Sustained funding is critical to support early childhood education and care programs, which helps recruit and retain trained educators who can optimize the cognitive and socioemotional development of the youngest generation (Organization for Economic Cooperation and Development, 2019).

The difficulty in increasing educators’ wages may constitute why there continue to be major gaps in this area of research. First, no known experimental or observational research has been able to examine the effect of sustained increased hourly wages on educators’ stress, particularly without workload increases (Otten et al., 2019). There has also been no known research to examine the relationship between educators’ wages and their physiological stress (Wettstein et al., 2021). This distinction is important because physiological stress levels may be different from one’s psychological perception of stress (Bärtl et al., 2023), and physiological stress is theorized as a major contributor to chronic disease later in life (Juster et al., 2010). Hence, our objective was to fill the gaps in this research by examining if wage increases were associated with changes in educators’ psychological and physiological stress.

## Theoretical Background and Definitions Related to Educators’ Wages and Stress

Inspiration for this study comes from two theories. The first is Kwon and colleagues’ (2021) educator-adapted Job Demands and Resources (JD-R) framework (Bakker & Demerouti, 2007), and the second is the allostatic load theory of stress (e.g., McEwen & Wingfield, 2010).

According to the educator-adapted JD-R framework (Kwon et al., 2021), when job demands (e.g., workload) exceed job resources (e.g., wages), educators must expend their own resources to manage this mismatch (Kwon et al., 2021). This expense may result in severe stress (Bakker & Demerouti, 2007; McEwen & Wingfield, 2010).

The allostatic load theory of stress delineates the necessity of stress for survival, but explains how differential stress responses to repeated stressors may eventually result in “wear and tear” on the body. To clarify, stress is the body’s psychological and physiological reaction to a perceived stressor. Psychological stress may reflect symptoms of perceived stress, anxiety, and depression. Physiological stress is often measured with biomarkers, such as cortisol (Russell et al., 2012). Cortisol is an end-product of one of the major stress response systems, the hypothalamus pituitary adrenal axis (HPA) axis, and helps regulate normal physiology, including in response to stressors.

According to the allostatic load theory, psychological and physiological stress responses may be counterbalanced to maintain the body in a type of homeostasis (McEwen & Wingfield, 2010). In other words, psychological stress levels may not directly align with physiological stress levels. For example, while one may perceive the absence of stress, physiological biomarkers may be high; similarly, one may perceive intense stress, but physiological biomarkers may be high or even low (Ford et al., 2019; Khoury et al., 2019). Stress responses may also depend on timing and one’s previous experiences. For educators, the anticipation and hectic preparation for the new academic year may prompt a heightened stress response, which may decrease after habituation (von der Embse & Mankin, 2021). In other instances, stress responses may be blunted due to occupational burnout, which includes feelings of exhaustion, depersonalization, and reduced personal accomplishment (Bärtl et al., 2023). For example, Bärtl et al. (2023) found no cross-sectional relationship between workplace burnout and physiological stress (measured with hair cortisol concentration). Yet, they found that higher burnout at time one predicted lower physiological stress at time two (Bärtl et al., 2023). In the Bärtl et al. (2023) study, the participants’ physiological stress response may have been exhausted from managing chronic psychological work-related stress, resulting in “wear and tear” on the body, also known as allostatic load (McEwen & Stellar, 1993).

### Empirical Work Related to Educators’ Wages and Stress

For context, a recent international systematic review and meta-analysis showed that large and relative wage increases may matter the most for mental health (*n* = 54 studies with nearly one million participants; Thomson et al., 2022). Thomson et al. (2022) found that while a 10% increase in income resulted in about 0.003 *SD* improvement in health, the effects were about 0.13 *SD* greater when individuals moved out of poverty (Thomson et al., 2022). When considering this research in the context of the United States, the federal poverty threshold has been argued to be inappropriately low for survival (Jiang et al., 2017). In 2023, the federal poverty threshold for a single adult across the 48 contiguous states was $14,580/year (Office of the Assistant Secretary for Planning and Evaluation, 2023). Instead, a livable wage threshold should be considered the threshold for a severe stressor. For a single adult with no children in Ohio, a livable wage is about $15.33/hour or nearly $32,000/year (Glasmeier, 2023).

Hence, the available investigations on educators’ hourly wages and stress may have been limited by inappropriate wage thresholds. For example, Kwon et al. (2021) found no significant cross-sectional association between educators’ wages and psychological stress. Besides the limitation of the cross-sectional analysis, the investigation into educators’ wages was further restricted by a dichotomous examination of an annual household income above and below $20,000/year (Kwon et al., 2021). This dichotomous threshold was much lower than what would be considered a “liveable wage” threshold for many adults in the United States, which may have accounted for the null findings.

In a larger cross-sectional mixed methods study, Otten et al. (2018) measured wages relative to the median hourly wage threshold of where educators worked. In Washington State and Texas, Otten et al. (2018) found that educators who earned below their locale’s median hourly wage had higher perceived stress than those who made above. However, there was no difference in educators’ depression. Besides the dichotomous examination of wages, focus groups revealed another major limitation. During the study, mandatory wage increases in Seattle unintentionally forced early educational centers to compensate for the lost income, such as by increasing classroom sizes and, consequently, educators’ workload (Otten et al., 2018). The educator focus groups mentioned that the increased workload was an added source of stress (Otten et al., 2018), which may have confounded the relationship between wages and stress.

### Moderating Influences on Educators’ Wages and Stress

The lack of examining moderating effects on the relationship between educators’ wages and stress may have also accounted for the previous findings. Moderators that should be prioritized include demographics (e.g., age, education, household income, race) and previous stressors. Demographics and exposure to previous stressors may influence educators’ access to material and mental resources that could help educators manage stressors and stress. For example, the combination of increased experience with age, and likelihood of earning higher wages over time, may strengthen the relationship between increased wages and decreased stress. Educators with higher education or higher household income may have increased knowledge-related resources and/or the likelihood of higher wages, which may increase their resilience or ability to access resources to manage stress. Race may also have a differential effect. For example, as compared to educators who are White, educators of minoritized races may experience differential societal stressors that could have limited their access to wealth (Alexander, 2010; Boyd-Swan & Herbst, 2019; Hamilton & Logan, 2020; Schaeffer, 2020) and increased their risk of stress (Bailey et al., 2017; Ford & Stowe, 2013, 2017). Finally, educators who have experienced significant previous stressors, such as adverse childhood experiences, may be at increased risk of stress (Anda et al., 2006). Previous stressors, or adverse childhood experiences, are increasingly considered a significant risk factor for vulnerability to future stress and stress-related disease. For educators with higher previous stressors or adverse childhood experiences, wages may have a decreased impact on their current stress.

### Unique Historical Influences on Educators’ Stress – The COVID-19 Pandemic

Difficulties examining the pay and stress for educators may also have been exacerbated by unique stressors during a monumental period in history - the COVID-19 pandemic. For historical context, the COVID-19 pandemic in the United States reached a critical point around March 2020, when many government leaders released “stay at home” orders (Office of Mike DeWine: Governor of Ohio, 2020). This stay-at-home order may have resulted in nearly 50% of educators worried that their money would not last, whereas, before the pandemic, 33% of educators had this worry (Swigonski et al., 2021). The pandemic also unexpectedly forced educators to the center of conflicts surrounding (a) mask and vaccine mandates for young children and (b) helping the children’s families navigate the hardships of the pandemic while managing their own (e.g., Souto-Manning & Melvin, 2022).

These COVID-19-related stressors may have resulted in an unprecedented workforce crisis. For example, in the Summer of 2021, a National Association for the Education of Young Children (NAEYC) survey of 7,500 educators showed that 80% of child care centers were understaffed (National Association for the Education of Young Children, 2021b). Further, more than 33% of educators were planning on either leaving the workforce or shutting down their childcare program, with more than 50% of “minority-owned programs” considering closing (National Association for the Education of Young Children, 2021b). About 80% of educators revealed that low pay was the reason for poor educator recruitment or why educators were leaving the field (National Association for the Education of Young Children, 2021b).

### The Current Study

This study innovatively addresses the prior research gaps surrounding educators’ wages and psychological and physiological stress in five ways. First, we address cross-sectional limitations by examining educators’ stress longitudinally across two time points. Second, we carefully consider several unique dimensions of educators’ psychological stress, including perceived stress, depression, and anxiety. We also examine educators’ anxiety with a clinically-based measure. Third, we examine chronic physiological stress in educators, including over time. We use a noninvasive and commonly used biomarker, hair cortisol concentration, to estimate educators’ chronic physiological stress. Fourth, examining dimensions of psychological stress with physiological stress allows the first examination of differential stress system responses in this population. Fifth and finally, we consider important moderators on the relationship between educators’ wages and stress.

We address these five research gaps through three specific aims. The first aim was to examine changes in teacher stress. We hypothesized that educators would habituate to stressors associated with the onset of the academic year and consequently have a later decrease in stress (both psychological and physiological). In other words, educators’ stress would be lower at TP2 compared to TP1. Our second aim was to examine if wage levels predicted changes in educators’ stress. We hypothesized that increases in hourly wages would predict decreased stress (both psychological and physiological). Our third aim was to examine moderating effects of demographics and previous stressor exposure on the relationship between wage changes and educators’ stress. We hypothesized that moderators representing increased access to material and mental resources would strengthen the relationship between increased wages and decreased stress.

## Methods

### Design, Participants, and Procedure

This study had a longitudinal, correlational design. Educators were recruited and enrolled in Fall 2021 with a targeted convenience sampling strategy from early educational centers around central and southwest Ohio. Emails and flyers were sent to administrators of early educational centers or listservs, with whom we had existing relationships. Educators were eligible to enroll if they were at least 18 years old and taught children 0–5 years old at an early childhood educational or care center. The University of Cincinnati Institutional Review Board (IRB) approved the study. After participant eligibility was confirmed, we collected electronic consent and data at educators’ convenience through online surveys and a hair sample.

Data from this study are from enrollment at time point (TP) 1 (September-October 2021) and TP2 (December 2021-February 2022). As a reminder, the unexpected emergence of the COVID-19 Omicron variant occurred around TP2. Further information about teacher participation is in Supplemental Fig. 1.

Brief sample characteristics are in Table 1 (details are in Bates & Dynia, 2023). At TP1, educators’ average age was 40 years old. About 95% of educators identified as female, and about 70% identified as White. Nearly 50% of educators had a bachelor’s degree or higher, and approximately 40% earned an annual household income of less than $30,000/year. At both time points, educators worked full-time (*p* = .64). Educators made about $16.36/hour at TP1, and earned nearly $2 more each hour at TP2 ($18.21; *p* = .007). Compared to educators who participated in both time points, educators who only participated in TP1 were younger (*p* = .016) and were more likely to not have a bachelor’s degree (*p* = .006; see Supplemental Table 2).

**Table 1:** Sociodemographic Characteristics of Early Childhood Educators and Main Variable Descriptives

	TP1 (total *n* = 67)							TP2 (total *n* = 53)							Change between TPs				
	*n*	%	*M*	*SD*	Range	α		*n*	%	*M*	*SD*	Range	α		*n*	*M*	*SD*	Cohen’s *d*	*p*
Age ^a^	65		39.43	13.47	19–69			52		41.42	12.56	22–69							
Hourly wages	57		$16.36	$4.39	$4.00-29.16			45		$18.21	$5.18	$11.80-18.21							
Weekly hours worked	64		38.08	9.76	5–60			53		39.43	7.17	12–55							
Previous stressors (ACES) ^a^	61		2.77	2.85	0–9			51		2.82	2.76	0–9							
Gender ^a^	63							51											
Female	60	95.2						48	94.1										
Male	3	4.8						3	5.9										
Education ^a^	65							52											
No bachelor’s degree	33	50.8						22		42.3									
Bachelor’s degree or higher	32	49.2						30		57.7									
Annual household income	63							52											
< $30,000/year	24	38.1						16	30.8										
>$30,001/year	39	61.9						36	69.2										
Race ^a^	67							53											
White	46	68.7						38		71.7									
Minoritized	21	31.3						15		28.3									
Stress																			
Physiological stress (log10 HCC pg/mg)	43		0.90	0.49	0.05–2.30			40		0.95	0.40	0.33–2.04			33	0.03	0.47	0.07	0.71
Perceived stress	61		20.74	7.44	4–34	0.88		53		19.81	6.39	34 − 32	0.88		51	-1.04	5.26	-0.20	0.17
General anxiety	61		8.03	5.97	0–21	0.93		53		7.68	5.27	0–21	0.90		51	-0.41	5.36	-0.08	0.59
Depression	60		20.00	11.90	2–46	0.91		53		18.92	11.40	2–45	0.92		51	-1.25	8.56	-0.15	0.15
Psychological stress composite	61		0	2.77	-4.54-5.78			53		0	2.70	-4.98-5.80			51	0.32	2.06	0.16	0.91

*Note* TP1 = time point 1; TP2 = time point 2; ACES = adverse childhood experiences; HCC = hair cortisol concentration. α = Cronbach’s alpha. Non-imputed TP1 HCC (in pg/mg) *M* = 16.51, *SD* = 32.13 (Range 1.11–199.83); Non-imputed TP2 HCC *M* = 15.41, *SD* = 22.23 (Range 2.14-110.33). Median wages at TP1 were $15.40/hour and at TP2 were $16.00/hour. Dependent samples *t*-tests then calculated with equal variances assumed and two-sided *p*-value, with Cohen’s *d* reported as a measure of effect

^a^ Data collected only in TP1

### Measures

Participants answered online survey questions on psychological stress (perceived stress, anxiety, and depression) and demographics, collected and managed with Qualtrics (Provo, UT) and research electronic data capture (REDCap) software (Harris et al., 2009, 2019). Willing participants with at least 1 cm length of hair provided hair samples to approximate physiological stress. Psychological and physiological stress were measured at both time points. Descriptives, including Cronbach’s alpha for some applicable measures, of main variables by time point are in Table 1.

### Psychological Stress

#### Perceived Stress

The Perceived Stress Scale (Cohen et al., 1983) was used to evaluate educators’ perceived stress based on experiences over the past month. Participants rated 10 situations on a five-point frequency scale (0 = *never* to 4 = *very often*). Scores were summed so that higher scores signify higher perceived stress (range 0–40). National means for women range from 13.7 (Cohen, 1994) to 16.14 (Cohen & Janicki-Deverts, 2012). In a study of educators, scores from 0 to 13 were considered “low stress,” 14–26 were considered “moderate stress,” and scores of 27–40 were considered “high stress” (Quinn et al., 2022). Higher scores on the Perceived Stress Scale are associated with increased symptoms of stressor-elicited depression (Cohen, 1994). Test-retest reliability is *r* = .85 between two days (Cohen et al., 1983).

#### Anxiety

Anxiety was measured over the last two weeks with the General Anxiety Disorder-7 scale (Spitzer et al., 2006). Participants rated seven items on a four-point frequency scale (0 = *not at all* to 3 = *nearly every day*). Scores were summed so that higher scores indicated higher anxiety (range 0–21). A score of 10 or greater has high sensitivity (89%) and specificity (82%) for a clinical diagnosis of generalized anxiety disorder (Spitzer et al., 2006). Test-retest reliability was 0.83 (Spitzer et al., 2006).

#### Depression

Depression was measured over the past week with the Center for Epidemiologic Studies Depression Scale (Radloff, 1977). Educators rated 20 items using a three-point frequency scale (0 = *rarely or none of the time, less than 1 day* to 3 = *most or all of the time, 5–7 days*). Scores were summed so that higher scores indicate higher depression symptoms (range 0–60). A score of 16 or greater for women with low income had high sensitivity (95%), specificity (70%), and negative predictive value (99.1%; positive predictive value of 28.4%) for a clinical diagnosis of major depressive disorder (Thomas et al., 2001). The correlation between the CES-D scores and observational ratings of depression were *r* = .56 (Radloff, 1977). The two-week test-retest reliability was *r* = .51 (Radloff, 1977).

### Physiological Stress

Physiological stress was estimated with hair cortisol concentration. Research staff cut a shoelace-tip-diameter hair sample next to the scalp skin, primarily from the posterior vertex. Lab personnel cut hair strands to 3 cm length from the scalp to represent the prior 3 months of hair growth from the sampling time (Loussouarn et al., 2016). Because we sampled hair at nearly the same time as the survey data, hair cortisol values represented 3 months of average physiological stress *preceding* participants’ psychological stress measures. Hair samples were then stored at room temperature and were analyzed for cortisol content with Salimetrics® (n.d.) immunoassay kit following established methods (Meyer et al., 2014). Samples were assayed in duplicate. The average intra-assay coefficient of variation (CV) was 6.7%, and the inter-assay CV was 6.0% (< 10% is considered acceptable). One value at TP1 and one value at TP2 were considered outliers at more than 3 *SD* above the sample mean. Due to a skewed distribution and in line with most published research with hair cortisol, hair cortisol values were transformed from pg/mg to log10 (pg/mg) to normalize the distribution and reduce the impact of the outliers on results.

No established cutoffs of hair cortisol concentration suggest a participant would have diagnostically “high” or “low” stress. However, there is published research that allows hair cortisol concentration comparisons. In a sample of primary caregivers living in poverty (mothers; *n* = 75; mean age 28.7 years), Bates et al. (2021) found a mean HCC (analyzed with immunoassay) of 6.18 pg/mg (*SD* = 7.04; range 0.10–31.26). In a sample of working women in Budapest and London (*n* = 164; mean age 43.6 years, *SD* = 9.8), Serwinski et al. (2016) found a mean HCC (analyzed with liquid chromatography-mass spectrometry) of 8.39 pg/mg (*SD* = 6.3). Finally, HCC values from “healthy” volunteers across four international laboratories ranged from 20.0 to 46.1 pg/mg (Albar et al., 2013).

Along with hair cortisol concentration collection, participants completed an established survey on major confounders of the measure (Bates et al., 2020). Steroid or hormone use emerged as a significant correlate of hair cortisol, but was used by about 50% of the sample (Bates et al., in review), so it was included as a covariate in the analysis.

### Covariates

Covariates were selected based on prior research and the characteristics of the sample. Covariates included demographics (participant race, annual household income, highest level of education, and age at enrollment) and significant stressor exposure (adverse childhood experiences [ACES]). We added one additional control for hair cortisol analyses - steroid or hormone use (e.g., pharmacologic birth control, steroid creams used for eczema or mosquito bites).

#### Demographics

Race was represented as a dichotomous variable (1 = *White*, 0 = *not White/ minoritized race including Black, Native Hawaiian or Other Pacific Islander, Asian, or Other*). This scoring decision was made as the differential racial or ethnic composition of teachers of color in the sample was small (*n* = 20 Black/African American; 1 = Native Hawaiian or Other Pacific Islander; 1 = Asian, 2 = Hispanic/Latino).

We controlled for annual household income at TP2 because we were primarily interested in changes in stress at TP2 (we included TP1 annual household income in the correlations, found in Table 2, for informational purposes). Annual household income was collected as categories of about $10,000 and was dichotomized as 0 = *≤$30,000/ year* and 1 = *> $30,000/year.* This cutoff was selected because it is the approximate livable wage cutoff for a single adult (with no children) working full-time in Ohio (Glasmeier, 2023).

**Table 2:** Correlations between Variables (Non-Imputed Data; *n* = 67)

	1	2	3	4	5	6	7	8	9	10	11	12	13	14	15	16	17	18	19	20
1. Age	--																			
2. Previous stressors (ACEs)	-0.11	--																		
3. Education	0.06	0.01	--																	
4. Race White	-0.19	0.26*	0.50**	--																
5. TP1 hourly wages	0.26*	0.20	0.42**	0.28*	--															
6. TP2 hourly wages	0.29	0.24	0.24	0.08	0.75**	--														
7. TP1 household income	0.30*	0.03	0.38**	0.23	0.39**	0.21	--													
8. TP2 household income	0.24	0.13	0.44**	0.31*	0.47**	0.43**	0.78**	--												
9. TP1 Physiological stress	0.24	0.12	-0.14	-0.09	-0.19	0.32	0.18	0.25	--											
10. TP2 Physiological stress	0.24	-0.11	0.02	-0.22	-0.10	0.15	-0.06	0.00	0.29	--										
11. Change in physiological stress	-0.11	0.14	-0.06	-0.19	0.06	-0.19	-0.20	− 0.36*	− 0.75**	0.42*	--									
12. TP1 Perceived stress	-0.15	0.30*	-0.14	0.20	0.06	0.13	0.08	0.06	-0.12	0.26	0.29	--								
13. TP2 perceived stress	-0.16	0.10	-0.21	-0.01	0.08	0.05	-0.02	-0.10	-0.05	0.27	0.23	0.73**	--							
14. TP1 anxiety	-0.15	0.36**	-0.04	0.30*	0.12	0.26	0.17	0.22	-0.07	0.28	0.15	0.82**	0.64**	--						
15. TP2 anxiety	0.05	0.22	-0.22	-0.02	-0.03	0.27	0.00	-0.02	0.09	0.06	0.09	0.61**	0.69**	0.59**	--					
16. TP1 depression	-0.11	0.34**	-0.08	0.27*	-0.03	0.20	0.08	0.11	−0.03	0.30	0.13	0.84**	0.57**	0.79**	0.56**	--				
17. TP2 depression	-0.08	0.26	-0.13	-0.03	0.07	0.28	0.02	-0.03	-0.02	0.04	0.15	0.66**	0.72**	0.60**	0.76**	0.75**	--			
18. TP1 composite of psychological stress	-0.14	0.36**	-0.09	0.27*	0.05	0.21	0.12	0.14	-0.08	0.30	0.20	0.95**	0.69**	0.93**	0.62**	0.94**	0.71**	--		
19. TP2 composite of psychological stress	-0.07	0.21	-0.21	0.00	0.05	0.23	0.00	-0.06	0.01	0.14	0.17	0.74**	0.89**	0.67**	0.90**	0.69**	0.92**	0.75**	--	
20. Change in composite psychological stress	0.09	-0.18	-0.04	− 0.33*	-0.05	0.01	-0.22	0.27	0.11	-0.27	0.06	− 0.36*	0.22	− 0.42**	0.34*	− 0.40**	0.23	− 0.42**	0.29*	--

*Note* TP = time point

* Correlation significant at the 0.05 level (2-tailed)

** Correlation significant at the 0.01 level (2-tailed)

Based on characteristics of the sample (details found in Bates & Dynia, 2023), educators’ highest education level was collapsed to: *no college degree, some college, or associate degree* (*n* = 33) and *Bachelor’s degree or higher* (*n* = 32). We also approximated educators’ age at enrollment by subtracting birth year from 2021 (when participants completed enrollment surveys). Based on the sample characteristics, we did not control for gender (only three participants identified as male) or ethnicity (only two participants identified as Latine).

#### Previous Stressor Exposure

The number of previous significant stressors was estimated with the deidentified count adaptation (Felitti et al., 1998) of adverse childhood experiences (Center for Youth Wellness, 2015). Participants indicated how many of 10 adverse childhood experiences they were exposed to before they were 18 years old. Briefly, these included a household member(s) (1) separated or divorced; (2) served time in jail or prison; (3) had a mental illness; (4) hurt or threatened to hurt each other; (5) acted in a way that made participant fearful; (6) touched or asked to touch participant’s “private parts” in an unwanted way; (7) went without food, clothing, place to live, or had no one for protection; (8) pushed, grabbed, slapped, or threw something at participant; (9) had a problem with drinking or using drugs; (10) did not support, love, or protect participant (Felitti et al., 1998). A score of four or greater indicates high risk for chronic disease (Felitti et al., 1998; Wade et al., 2017).

### Analytic Overview

Before enrolling the first participant, we calculated power for this pilot study in G*Power version 3.1.9.1 (Faul et al., 2007). With two tails, an alpha of 0.05, and power at 0.80, we had the power to detect (a) moderate effects at *dz* = 0.35 for differences between two dependent means and (b) small effects at *f*^2^ = 0.12 for seven regression predictors.

We conducted the analyses through four primary steps. First we examined direct changes in teacher stress over two time points with dependent-samples *t*-tests. Second, we used correlations to examine how changes in hourly wages were directly associated with changes in psychological and physiological stress. Third, we examined multiple linear regressions to predict changes in educators’ psychological and physiological stress, controlling for covariates. In line with guidelines on regression-based change score analysis (Allison, 1990; Mattes & Roheger, 2020), the dependent variable change scores were created by subtracting the TP1 stress variable from the TP2 stress variable; the TP1 stress variable was also added as a covariate in the regression analyses (a covariate that should not be interpreted). Fourth, we explored how demographics (age, education, household income, race) and previous stressors moderated the effect of wages on changes in educators’ physiological and psychological stress.

Because of limited power and because the psychological stress variables (depression, anxiety, perceived stress) were often highly correlated with each other (see Table 2), we created a psychological stress composite variable for the analyses in Aim 2 and 3. The psychological stress composite was created by summing raw (non-imputed) *z*-scores ([*x* - Mean of the sample]/ *SD* of the sample) of perceived stress, depression, and anxiety for each time point. The change score was then created by subtracting the TP1 stress composite variable from the TP2 stress composite variable.

As with most observational longitudinal data in social sciences, we had some missing data (see Supplemental Table 1 for details). Up to 33% of data were missing for survey variables and up to 40% for physiological stress variables. Little’s Missing Completely at Random (MCAR) test was not significant (Chi-Square = 233.730, *df* = 208, *p* = .107), indicating that we failed to reject the null hypothesis that data were MCAR. Because analyzing only complete cases would represent those who *chose* to complete full measures, we followed guidelines to decrease this participation bias and improve power (Woods et al., 2021, 2023) by multiply imputing for all variables. Following these guidelines (Woods et al., 2021, 2023), we performed all variable transformations before imputation (e.g., psychological stress composite, interaction variables for the moderation). We then multiply imputed missing variables in 20 datasets with the automatic method in SPSS (fully conditional specification). Variables used in the multiple imputation were 16 variables used in some form in the regression analyses. These variables included the (1) physiological stress change score, (2) physiological stress in TP1, (3) psychological stress change score, (4) psychological stress in TP1, (5) wage change score, (6) household income in TP2, (7) previous stressors (ACES), (8) education, (9) race, (10) age, (11) hormone or steroid use in TP2, and interaction variables (wages with [12] previous stressors, [13] education, [14] income, [15] race, and [16] age). We provide regression analyses without multiple imputations in Supplemental Tables 3, 4, and 5 for informational purposes. However, readers should be cautioned about interpreting the non-imputed results because of the low sample size.

## Results

### Preliminary Results

Here, we describe educators’ wages and direct associations between stress and demographic variables with results from Table 1 (descriptive statistics) and Table 2 (correlations). Table 1 shows that the mean wage increase of the educators was about $1.85/hour, but the median wage increase was about $0.40/hour. A separate analysis showed that few educators had a change in their approximately “livable” annual household income level in Ohio for a single adult with no children from TP1 to TP2. That is, across TP1 to TP2, three educators increased their total household annual income from ≤ $30,000 to > $30,000, while three others had a decrease from > $30,000 to ≤ $30,000.

Next, we examined significant correlations among psychological and physiological stress variables in Table 2. Psychological stress variables were strongly correlated with each other. Physiological stress at TP2 was only marginally correlated with TP1 depression (*r* = .30, *p* = .06, *n* = 38). In other words, educators’ physiological stress levels *preceding* the start of the academic year (TP1) were not related to psychological stress at the start of the academic year or later (TP1 and TP2). Instead, educators’ depression at the start of the academic year (TP1) was marginally moderately associated with higher physiological stress levels (TP2) over about the next three months. Finally of note, the only other variable directly associated with psychological stress was race. Compared to educators of a minoritized race or ethnicity, educators who were White tended to have a moderate decrease in psychological stress (*r* = − .33, *p* = .02, *n* = 51).

We then examined significant correlations between educators’ wages and their demographics. Higher wages at TP1 were associated with identifying as White (as compared to a minoritized race; *r* = .28, *p* = .04, *n* = 57), increased age (*r* = .26, *p* = .049, *n* = 57), higher education (*r* = .42, *p* < .001, *n* = 57), and higher household income (TP1 *r* = .39, *p* < .001, *n* = 56; TP2 *r* = .47, *p* < .001, *n* = 45). Higher wages at TP2 were only significantly associated with higher household income at TP2 (*r* = .43, *p* < .001, *n* = 44).

We also noted additional disparities in our sample. Higher education was positively associated with White race (*r* = .50, *p* < .001, *n* = 65) and higher household income (TP1 *r* = .38, *p* < .001, *n* = 63; TP2 *r* = .44, *p* < .001, *n* = 51). Higher household income was significantly and positively associated with White race at TP2 (TP1 *r* = .23, *p* = .07; TP2 *r* = .31, *p* = .03). We then probed the racial disparities correlations with a post-hoc analysis. Regarding race and hourly wages, at TP1, minoritized educators earned $14.50/hour (*SD* = 4.82), while White educators earned significantly more at $17.15/hour (*SD* = 4.00; *t* = -2.15, *p* = .036, equal variances assumed independent samples *t*-test). At TP2, this wage difference tapered and was no longer significant (minoritized *M* = $17.56/hour, *SD* = 7.26; White *M* = $18.47/hour, *SD* = 4.18, *t* = -0.53, *p* = .597). Regarding race and earning a livable household income, there was no significant association between race and livable annual household income at TP1 (χ2 [1] = 3.23, *p* = .07, *n* = 63), rather a significant association at TP2 (χ2 [1] = 5.04, *p* = .3, *n* = 52). Specifically, the odds of earning less than $30,000/year at TP2 for minoritized individuals was 4.14 times the odds among those who were White (95% CI 1.15, 14.92). Finally, regarding race and education, we found a significant association of education and race (χ2 [1] = 16.09, *p* < .001, *n* = 65) in that the odds of not obtaining a bachelor’s degree for minoritized educators was 15.94 times the odds of White educators (95% CI 3.26, 77.83).

### Aim 1: Examine Changes in Teacher Stress

Table 1 shows that educators’ stress symptoms did not significantly and directly change between the time points. To probe this relationship, we distinguished individuals by diagnostic levels of anxiety (score of 10 or greater on the General Anxiety Disorder-7 scale; Spitzer et al., 2006), depression (score of 16 or greater on the Center for Epidemiologic Studies Depression Scale; Thomas et al., 2001), and perceived stress from a previous sample of educators (scores > 13 on the Perceived Stress Scale indicating moderate to high stress; Quinn et al., 2022). Results showed that about 41% of educators at TP1 and 34% at TP2 met the criteria for generalized anxiety disorder. Further, 58% of educators at TP1 and 55% at TP2 met the criteria for major depressive disorder. Finally, about 82% of educators at TP1 and 83% at TP2 met criteria for moderate to severe stress. Changes in established cutoff levels of stress weren’t significantly different (McNemar’s test *p* > .05). In conclusion, in contrast to our hypothesis, educators’ stress did not significantly and directly change.

### Aim 2: Examine if Wage Levels Predicted Changes in Teacher Stress

To test Aim 2, we examined direct correlations and multiple linear regressions. According to the correlations in Table 2, TP1 wages and wage changes were not significantly and directly associated with most aspects of educators’ stress. However and unexpectedly, higher TP2 wages were moderately, marginally significantly associated with higher TP2 depression (*r* = .28, *p* = .06). We then tested these relationships with controls using multiple linear regressions, shown in Table 3. In contrast to our hypothesis, wage changes did not significantly predict changes in educators’ physiological or psychological stress.

**Table 3:** Regressions Predicting Physiological and Psychological Stress in Early Childhood Educators (Imputed Data, *n* = 67)

	DV: Change in Physiological Stress							DV: Change in Psychological Stress					
	Unstandardized				95% *CI*			Unstandardized				95% *CI*	
	*b*	*SE*	*t*	*p*	*LB*	*UB*		*b*	*SE*	*t*	*p*	*LB*	*UB*
Constant	0.45	0.41	1.12	0.27	-0.37	1.28		0.67	1.35	0.50	0.62	-2.01	3.35
Wage change	0.00	0.03	0.08	0.94	-0.06	0.06		-0.06	0.13	-0.47	0.64	-0.32	0.20
TP2 household income	-0.23	0.22	-1.08	0.29	-0.67	0.20		-0.57	0.89	-0.64	0.52	-2.33	1.20
Previous stressors (ACES)	0.03	0.03	1.14	0.26	-0.02	0.08		0.06	0.11	0.56	0.58	-0.16	0.28
Education	0.21	0.16	1.31	0.19	-0.11	0.54		0.55	0.76	0.72	0.47	-0.96	2.06
Race White	-0.21	0.22	-0.97	0.34	-0.64	0.23		-1.38	0.82	-1.68	0.10	-3.00	0.24
Age	0.01	0.01	0.93	0.36	-0.01	0.02		0.01	0.03	0.22	0.83	-0.05	0.06
TP2 hormone or steroid use	0.20	0.14	1.38	0.17	-0.09	0.48							
TP1 physiological stress	-0.73	0.21	-3.52	0.00	-1.16	-0.31							
Stress Composite TP1								-0.22	0.11	-1.94	0.05	-0.44	0.00

*Note* DV = dependent variable; CI = confidence interval; LB = lower bound; UB = upper bound; ACES = adverse childhood experiences

### Aim 3: Examine Moderating Effects on Wage Levels to Predict Teacher Stress

The moderating effects of demographics and previous stressors on wage changes predicting stress are in Table 4 (dependent variable physiological stress) and Table 5 (dependent variable psychological stress). In contrast to our hypothesis, there were no significant moderating effects of wage changes on changes in educators’ physiological stress or psychological stress.

**Table 4:** Moderating effects of wage change on changes in physiological stress in early childhood educators (Imputed Data, *n* = 67)

	DV: Change in Physiological Stress					
	Unstandardized				95% *CI*	
	*b*	*SE*	*t*	*p*	LB	UB
(Constant)	0.50	0.40	1.24	0.22	-0.31	1.31
Wage change x age	0.00	0.00	0.41	0.68	0.00	0.00
Wage change	-0.03	0.08	-0.32	0.75	-0.18	0.14
TP2 household income	-0.22	0.22	-0.96	0.34	-0.67	0.24
Previous stressors	0.03	0.03	1.21	0.23	-0.02	0.09
Education	0.22	0.17	1.31	0.19	-0.11	0.56
Race White	-0.22	0.22	-0.98	0.33	-0.67	0.23
Age	0.00	0.01	0.66	0.51	-0.01	0.02
TP2 hormone or steroid use	0.19	0.14	1.34	0.18	-0.09	0.47
TP1 physiological stress	-0.74	0.21	-3.52	0.00	-1.16	-0.31
(Constant)	0.48	0.39	1.23	0.23	-0.31	1.26
Wage change x previous stressors	-0.01	0.01	-0.97	0.33	-0.03	0.01
Wage change	0.03	0.04	0.62	0.54	-0.06	0.11
TP2 household income	-0.22	0.21	-1.03	0.31	-0.65	0.21
Previous stressors	0.04	0.03	1.31	0.20	-0.02	0.09
Education	0.22	0.16	1.34	0.18	-0.10	0.54
Race White	-0.19	0.22	-0.86	0.39	-0.63	0.25
Age	0.00	0.01	0.74	0.46	-0.01	0.01
TP2 hormone or steroid use	0.16	0.15	1.09	0.28	-0.11	0.47
TP1 physiological stress	-0.73	0.21	-3.45	0.00	-1.17	-0.30
(Constant)	0.47	0.38	1.23	0.23	-0.30	1.23
Wage change x education	-0.02	0.07	-0.22	0.82	-0.15	0.12
Wage change	0.01	0.05	0.20	0.84	-0.09	0.11
TP2 household income	-0.23	0.22	-1.06	0.30	-0.67	0.21
Previous stressors	0.03	0.03	1.11	0.27	-0.02	0.08
Education	0.23	0.17	1.36	0.18	-0.10	0.56
Race White	-0.21	0.21	-1.00	0.32	-0.63	0.21
Age	0.01	0.01	0.83	0.41	-0.01	0.02
TP2 hormone or steroid use	0.19	0.16	1.21	0.23	-0.12	0.50
TP1 physiological stress	-0.73	0.21	-3.59	0.00	-1.15	-0.32
(Constant)	0.45	0.40	1.14	0.26	-0.35	1.25
Wage change x income	0.01	0.05	0.12	0.91	-0.09	0.11
Wage change	0.00	0.04	-0.01	0.99	-0.08	0.08
TP2 household income	-0.24	0.22	-1.09	0.28	-0.68	0.20
Previous stressors	0.03	0.03	1.12	0.27	-0.02	0.08
Education	0.21	0.17	1.28	0.21	-0.12	0.54
Race White	-0.21	0.22	-0.97	0.34	-0.64	0.23
Age	0.01	0.01	0.96	0.34	-0.01	0.02
TP2 hormone or steroid use	0.20	0.15	1.38	0.17	-0.09	0.49
TP1 physiological stress	-0.73	0.21	-3.46	0.00	-1.17	-0.30
(Constant)	0.47	0.39	1.19	0.24	-0.33	1.26
Wage change x race	-0.04	0.06	-0.74	0.46	-0.16	0.07
Wage change	0.02	0.04	0.51	0.61	-0.06	0.10
TP2 household income	-0.22	0.22	-0.99	0.33	-0.66	0.23
Previous stressors	0.03	0.03	1.22	0.23	-0.02	0.08
Education	0.20	0.17	1.22	0.23	-0.13	0.53
Race White	-0.16	0.24	-0.69	0.50	-0.64	0.32
Age	0.00	0.01	0.66	0.51	-0.01	0.02
TP2 hormone or steroid use	0.18	0.14	1.24	0.22	-0.11	0.47
TP1 physiological stress	-0.72	0.21	-3.46	0.00	-1.15	-0.30

*Note* CI = confidence interval; LB = lower bound; UB = upper bound; x = interaction; TP = time point

**Table 5:** Moderating Effects of Wage Change on Changes in Psychological Stress in Early Childhood Educators (Imputed Data, *n* = 67)

	DV: Change in Psychological Stress					
	Unstandardized				95% *CI*	
	*b*	*SE*	*t*	*p*	LB	UB
(Constant)	1.06	1.39	0.76	0.45	-1.70	3.82
Wage change x age	0.01	0.01	0.90	0.37	-0.01	0.02
Wage change	-0.42	0.41	-1.02	0.31	-1.22	0.39
TP2 household income	-0.36	0.95	-0.38	0.71	-2.26	1.54
Previous stressors	0.12	0.12	1.00	0.32	-0.11	0.34
Education	0.68	0.72	0.95	0.35	-0.73	2.09
Race White	-1.45	0.81	-1.80	0.07	-3.04	0.14
Age	-0.01	0.03	-0.28	0.78	-0.08	0.06
Stress composite TP1	-0.26	0.12	-2.11	0.04	-0.51	-0.02
(Constant)	0.64	1.36	0.47	0.64	-2.07	3.34
Wage change x previous stressors	0.01	0.05	0.29	0.77	-0.08	0.11
Wage change	-0.10	0.21	-0.49	0.63	-0.52	0.32
TP2 household income	-0.60	0.91	-0.66	0.51	-2.42	1.22
Previous stressors	0.05	0.12	0.41	0.69	-0.19	0.29
Education	0.58	0.77	0.75	0.46	-0.95	2.10
Race White	-1.45	0.86	-1.69	0.09	-3.15	0.24
Age	0.01	0.03	0.31	0.76	-.05	0.07
Stress composite TP1	-0.21	0.13	-1.70	0.09	-0.46	0.03
(Constant)	0.70	1.36	0.51	0.61	-2.00	3.39
Wage change x education	-0.09	0.26	-0.35	0.73	-0.61	0.43
Wage change	-0.02	0.20	-0.09	0.93	-0.42	0.38
TP2 household income	-0.53	0.92	-0.58	0.57	-2.38	1.32
Previous stressors	0.07	0.11	0.64	0.52	-0.15	0.30
Education	0.68	0.79	0.87	0.38	-0.86	2.23
Race White	-1.42	0.84	-1.69	0.09	-3.08	0.23
Age	0.00	0.03	0.10	0.92	-0.06	0.07
Stress composite TP1	-0.23	0.12	-1.98	0.05	-0.46	0.00
(Constant)	0.63	1.38	0.46	0.65	-2.12	3.37
Wage change x income	0.03	0.26	0.13	0.90	-0.48	0.55
Wage change	-0.08	0.20	-0.40	0.69	-0.48	0.32
TP2 household income	-0.62	1.03	-0.60	0.55	-2.68	1.44
Previous stressors	0.06	0.11	0.55	0.58	-0.16	0.28
Education	0.57	0.74	0.76	0.45	-0.90	2.03
Race White	-1.37	0.83	-1.65	0.10	-3.01	0.27
Age	0.01	0.03	0.25	0.80	-0.06	0.07
Stress composite TP1	-0.22	0.12	-1.88	0.06	-0.45	0.01
(Constant)	0.68	1.36	0.50	0.62	-2.01	3.38
Wage change x race	-0.11	0.27	-0.42	0.68	-0.64	0.42
Wage change	-0.01	0.18	-0.07	0.95	-0.37	0.35
TP2 household income	-0.52	0.92	-0.56	0.58	-2.35	1.32
Previous stressors	0.08	0.11	0.66	0.51	-0.15	0.30
Education	0.52	0.76	0.69	0.49	-0.98	2.02
Race White	-1.25	0.87	-1.43	0.16	-2.97	0.48
Age	0.00	0.03	0.07	0.95	-0.06	0.07
Stress composite TP1	-0.23	0.12	-1.98	0.05	-0.47	0.00

*Note* CI = confidence interval; LB = lower bound; UB = upper bound; x = interaction; TP = time point

## Discussion

In this study, we analyzed changes in educators’ stress around the first half of the academic year 2021–2022, including if their change in hourly wages influenced changes in their stress. This study is one of the first to longitudinally and comprehensively examine educators’ stress – including anxiety and physiological stress measured with hair cortisol – with about 3 months occurring between two time points of measurement. Four major findings emerged. First, in contrast to our hypothesis, educators’ stress did not change but remained high over time. Second, in contrast to our hypothesis, wages did not predict changes in educators’ stress. Third, we found unique associations between educators’ physiological stress and depression symptoms, depending on the timing. That is, educators’ psychological depression symptoms at the start of the academic year were marginally positively related to their next three months of physiological stress. Fourth, we found evidence of race-based disparities in wages, as well as household income and education. Compared to educators who were White, educators of a minoritized race earned fewer hourly wages, were less likely to have a livable annual household income, and were less likely to have a bachelor’s degree. Here we interpret these four main findings within the context of previously published research, limitations of the nonexperimental nature of the study, and the extraordinarily rare and extenuating historical circumstances of the COVID-19 pandemic that may have affected our findings.

Regarding our first major finding, educators maintained high levels of stress from the start of the academic year to about three months later. For measures with evidence-based clinical cut points, we saw that about 1/3 of educators met clinically diagnostic levels of generalized anxiety disorder, and an alarming amount of over 1/2 of educators may have had diagnostic levels of depression. Further, 4/5 of educators had moderate to severe stress. These numbers are unprecedented. While these evaluations occurred during the COVID-19 pandemic, which increased stress among all adults across the United States (McKnight-Eily et al., 2021), the prevalence of depression among educators in this sample is double that of national intra-pandemic estimates (Ettman et al., 2020, 2022; Rosenberg et al., 2021). Further, the moderate to high perceived stress level of educators here was about 50% higher than that of a previous sample from Washington state in February/March 2021 (82% in this study compared to 57% in Quinn et al., 2022). These statistics illustrate the stress-influencing burden educators may have carried while educating children, maintaining classrooms, and, importantly, managing their own families and lives during the COVID-19 pandemic. Some of these burdens during 2021 included having chronically low wages despite decades of vocal advocacy (American Federation of Teachers, n.d.), which further threatened educators’ livelihood during a period of historic, sharp inflation (U.S. Bureau of Labor Statistics, 2022), and overwhelming concerns over the increased risk of severe illness and death for themselves and their loved ones (Whitaker et al., 2021). Educators’ inability to change these burdens and stressors also demonstrates, despite their essential role, their unjust lack of power in society. Educators’ persistent lack of power may be due to the United States’s culture of rewarding successful capitalist systems – a structure incompatible with early childhood education centered on equity – and oppression due to educators simply having to spend their valuable time working additional jobs to financially survive (Center for the Study of Child Care Employment, 2020).

Regarding our second major finding, hourly wage changes were not related to changes in educators’ stress. This nonsignificant finding may result from the potentially small difference in wage increase across the time points (Mean = $1.85/hour; Median = $0.40/hour) or due to study limitations (that perhaps contributed to a type II error). While the limitations of our study will be discussed in more depth later, the small difference in wage changes in our study needs interpretation here. In our longitudinal observational study, participants had a mean hourly wage increase of about $1.85/hour from TP1 to TP2. From another perspective, this wage increase equated to about $325/extra each month or about $3,900/year (working 40 h/week for 52 weeks). To put this wage increase into context for our sample, nearly 40% of our participants earned less than $30,000/year - an unlivable wage for a single adult with no children who is working full-time in Ohio (Glasmeier, 2023). Yet, few educators crossed the threshold of earning more than $30,000/year across the time points, and some even learned less over the time points. Further, we unfortunately did not collect information on the number of individuals in the educators’ households, which could have meant that educators needed an even higher income to be considered liveable. Regardless, this means that, despite the wage increase, the educators in our sample were still likely struggling to financially survive.

Further, the wage increase in this study was likely inadequate due to the unprecedented and historical increase in inflation during a unique time of the pandemic (U.S. Bureau of Labor Statistics, 2022). To recall, this study was conducted around September-October 2021 (TP1) and December 2021-February 2022 (TP2). Around this time, inflation was increasing; across the short study period itself, inflation jumped 2.5% (September 2021: 5.4%, February 2022: 7.9%; U.S. Bureau of Labor Statistics, 2022). In other words, the mean wages of $16.36/hour at the beginning of TP1 (September 2021) had a buying power of $16.92 by the end of TP2 (February 2022; U.S. Bureau of Labor Statistics, n.d.), suggesting that the wage increase of about $1.85/hour was only about $1.29/hour after adjusting for inflation. Further, the median wage increase was nearly negligent ($0.07/hour), after adjusting for inflation (U.S. Bureau of Labor Statistics, n.d.).

In addition to inflation, the academic year 2021–2022 occurred during a time of extraordinary housing costs (Bhattarai, 2022, February 10). The surge of the Omicron variant of COVID-19 around this time also resulted in the risk of lost wages or increased workload, as rampant illness again forced teacher absences and closings of classrooms (Richards, 2022, January 5). These factors also occurred during the major holiday season in the United States, where families are often expected to purchase additional food and gifts for many cultural and religious celebrations (e.g., Thanksgiving, Christmas). Thus, even with a slight increase in wages, it was simply extraordinarily difficult for educators to financially survive at this time. Coupled with educators’ inadequate wage increase, our nonsignificant finding aligns with the recent international systematic review by Thomson et al. (2022), who found that *large* and *relative* wage increases matter most for mental health.

The unique time of our study may have also influenced educators’ financial resources beyond wages. Although the study was pre-planned in early Spring 2021, we became aware of new government initiatives to help jumpstart the ailing early childhood education and care sector *during* data collection. For example, employment of child-care-related workers in Fall of 2021 was down 10% from pre-pandemic levels (Long, 2021, September 19), and 76% of daycare centers in Ohio were experiencing staffing shortages (National Association for the Education of Young Children, 2021a). To bring educators back into the workforce, local, state, and national initiatives focused on educators emerged around Summer/Fall 2021. For example, the City of Columbus, Ohio offered cash sign-on bonuses (NBC4 Staff, 2021), the Ohio state government offered at least $1000 of Hero Pay (Ohio Child Care Resource and Referral Association, 2021), and other Federal government assistance packages helped employers raise educators’ wages (Johnston, 2023, April 8). Other economic stimulus packages may have affected educators’ financial resources, such as child tax credits for those with minor dependents and loan pause payments for educators with college loans. While we did not collect specific information on the exact financial amounts that were supplementing educators’ wages and annual household income during this historic time, our study also shows that these stimulus packages were likely not enough to relieve educators’ stress. This is also critical to understand, as many educators in the United States also do not receive comprehensive health insurance as a result of variable employer-based benefits (Baldwin et al., 2007; Rudich et al., 2021), which should minimally include evidence-based screening, primary care treatment, and therapy.

Regarding our third major finding, there was evidence of a relationship between educators’ physiological stress and psychological depression symptoms, depending on the timing. That is, educators’ depression symptoms at the start of the academic year (TP1) were marginally positively related to their next three months’ of physiological stress (TP2). While this might be an incidental finding, this relationship aligns with the Bärtl et al. (2023) study, showing psychological occupational burnout predicts later physiological stress. This finding may also align with the allostatic load theory, in that the stressful nature of starting a new academic year may prompt the body to maintain a type of homeostasis. However, after time, the body may tire of managing chronic psychological stress, and effects may manifest as physiological stress, or other signals of “wear and tear” on the body.

Regarding our fourth major finding, we showed evidence that minoritized educators in central and southwest Ohio earned lower wages, had less livable annual household income, and were less likely to have a bachelor’s degree as compared to White educators. This finding aligns with the most recent national research available showing that Black early childhood educators earned $0.78 less/hour than their White colleagues in 2019 (Center for the Study of Child Care Employment, 2020) and that Black and Hispanic educators were less likely to have a bachelor’s degree than their White colleagues (Paschall et al., 2023). It is important to note that the reason for minoritized educators’ lower pay is not simply associated with their decreased likelihood of having a bachelor’s degree. That is because, unlike 96/98 majors studied, educators with an early childhood education bachelor’s degree are *unlikely* to have higher pay at the end of their career than educators *without degrees* (Broady & Hershbein, 2020). Further, cumulative lifetime earnings for those with a bachelor’s degree in early childhood education are the lowest of all majors (Broady & Hershbein, 2020). Besides earning the least of all majors (Broady & Hershbein, 2020) and working in an occupation that makes nearly the least (McLean et al., 2021), the wage gap for minoritized educators could equate to at least a $400,000 difference (not considering inflation) over a 40-year career ($0.78/hour = $1,622 earnings over one year invested with $1,622 added each year over 40 years with a 7.5% rate of return). Because bachelor’s degrees are costly and could further widen minoritized educators’ wage gap, it begs the question: what would be the incentive for minoritized educators to obtain a bachelor’s degree in early childhood education, especially when it is not often required in the United States?

Reasons for educators to obtain a bachelor’s degree may be personal, but the decision may unintentionally affect children. In addition to wage disparities, educators’ educational attainment disparities can be an unfair disadvantage for all children in their classrooms, especially minoritized children. Children of color benefit from having educators who look like them (National Association for the Education of Young Children & The Educational Trust, 2019). Educators who mirror the racial identity of the children in their classroom close race-based student achievement gaps (Dee, 2004), serve as important role models, and help break down harmful racist stereotypes (National Association for the Education of Young Children & The Educational Trust, 2019). However, teachers’ wages and educational attainment has a strong positive correlation with lower turnover, classroom quality, teacher-student relationships, and student outcomes (Bassok et al., 2021; Grunewald et al., 2022; Manning et al., 2017). As such, a bachelor’s degree is now the minimum qualification to be a teacher in early childhood education and care in the other 75% member countries of the Organization for Economic Co-operation and Development (OECD, 2019). Thus, if minoritized children do not have access to minoritized educators with equal education and pay as White educators, minoritized children are unjustly ripped of their right to an equal opportunity in education – particularly during the most critical period of brain development in their life (Shonkoff et al., 2000). Accordingly, eliminating race-based inequities in educator pay and education is a basic human rights issue not only for women and women of color who disproportionately comprise the educator workforce (McLean et al., 2021; Paschall et al., 2021), but also for the children in their classrooms.

### Limitations

During this study, there were five major design or historically unavoidable limitations that could have confounded our findings. First, there was a possibility of a type II error from null hypothesis significance testing (i.e., there may be an effect but we did not find one). One of the reasons for the null findings may be because we did not measure job-specific stress and biomarkers of stress may be inexact, as direct biomarkers of stress also represent normal physiology (see Bates et al., 2022 for a review). Additionally, we did not consider the influence of non-cash-related resources on teachers’ wages, such as the supplemental nutrition assistance program. Second, our study was nonexperimental (i.e., true causal findings can typically be determined with well-designed randomized controlled trials). Third, our study had low power (e.g., small sample size). The low power also precluded us from using a structural equation model with psychological stress as a latent variable. Fourth, the time between our measures may have been insufficient for detecting effects. Three months may have been insufficient for educators to habituate to the stressful start of the school year. Fifth, our study was conducted in one state of the United States, which may limit the generalizability of the findings. However, the findings may still be of international importance, as stress in educators is not just local to the United States. For example, stress may have influenced why 75% of Australian educators planned on leaving their position within the next three years (United Workers Union, 2021).

Nevertheless, this study helps draw important attention to racial wage and stress disparities in educators. We hope researchers take the information from this study, including the limitations, to design improved studies to improve the educator workforce – a benefit that would also improve the millions of families and children impacted by educators’ extraordinary and essential role in society.

### Future Research, Practice, and Policy Implications

Inspired by previous research and building upon the limitations of this study, future research, such as a high-quality randomized controlled trial, should investigate the effects of wage changes on other health and workplace outcomes of educators – such as turnover, workplace-related stress, and physical health. Research should also examine the effects over the course of the full academic year.

In line with other pre-and intra-pandemic studies, our research again showed that educators are likely experiencing high levels of stress (Elharake et al., 2022; Linnan et al., 2017; Otten et al., 2019; Swigonski et al., 2021; Whitaker et al., 2015). To adequately address the roots of these stress-related disparities, researchers, policymakers, and occupational health practitioners should design multi-level interventions and policies to help educators limit and manage their stress (Berger et al., 2022). From the micro- to macro-level, a few of these multi-level interventions should include person-based self-care, and use of psychotherapy approaches and perhaps prescription medications for mental health (micro-level); professional development, reflective coaching, positive professional relationships, and a manageable workload (workplace); affordable and easy *access* to psychotherapy and prescription medications to manage mental health (workplace and macro-level); and multi-level policies to eliminate wage-related disparities of educators and to minimally allow the primarily female and disproportionately of color workforce earn at least a livable wage (Berlin et al., 2020; Jennings et al., 2020; Rombaoa Tanaka et al., 2020; Sandilos et al., 2020).

## Conclusions

In this longitudinal observational study, we examined if wage increases were associated with stress reduction in a sample of educators during the 2021–2022 academic year - about one year after the COVID-19 pandemic began and during the surge in the highly contagious and disruptive Omicron variant. We found that educators’ stress remained persistently high and that wage increases were not associated with decreases in stress. However, racial disparities in educators’ wages were apparent in that minoritized educators earned less than White educators. This study will help researchers, practitioners, and policymakers design future work to improve the lives of educators – an essential undertaking that would also benefit all of society.

## Electronic Supplementary Material

Below is the link to the electronic supplementary material.

Supplementary Material 1

## Data Availability

Data are available upon request.
