# Supplementary material for: Changes in Stress Following Wage Increases for Early Childhood Educators
Source: Early Child Educ J. 2024 Apr 17;53(4):1195–213. doi: 10.1007/s10643-024-01666-0 (PMC11906561; doi:10.1007/s10643-024-01666-0)
Supplement: Supplementary file 1 — Supplementary Material 1 [file 10643_2024_1666_MOESM1_ESM.docx]

**Supplemental Table 1**

***Missing Data (TP1 n = 67, TP2 n = 53)***

| Variable | Complete data | # Missing by TP | % Missing by TP | # Missing by TP1 | % Missing by TP1 |
| --- | --- | --- | --- | --- | --- |
| Year born | 65 | 2/67 | 3 | 2/67 | 3 |
| Hourly wages TP1 | 57 | 10/67 | 15 | 10/67 | 15 |
| Hourly wages TP2 | 45 | 8/53 | 15 | 22/67 | 33 |
| Previous stressors (ACES) | 61 | 6/67 | 9 | 6/67 | 9 |
| Gender | 63 | 4/67 | 6 | 4/67 | 6 |
| Education | 65 | 2/67 | 3 | 2/67 | 3 |
| Annual household income TP1 | 63 | 4/67 | 6 | 4/67 | 6 |
| Annual household income TP2 | 52 | 1/53 | 2 | 15/67 | 2 |
| Race White | 67 | 0/67 | 0 | 0/67 | 0 |
| Physiological stress TP1 | 43 | 24/67 | 36 | 24/67 | 36 |
| Physiological stress TP2 | 40 | 13/53 | 28 | 27/67 | 40 |
| Perceived stress TP1 | 61 | 6/67 | 9 | 6/67 | 9 |
| Perceived stress TP2 | 53 | 0/53 | 0 | 14/67 | 0 |
| General Anxiety TP1 | 61 | 6/67 | 9 | 6/67 | 9 |
| General Anxiety TP2 | 53 | 0/53 | 0 | 14/67 | 21 |
| Depression TP1 | 60 | 7/67 | 10 | 7/67 | 10 |
| Depression TP2 | 53 | 0/53 | 0 | 14/67 | 21 |
| Food insecurity TP1 | 65 | 2/67 | 3 | 2/67 | 3 |
| Food insecurity TP2 | 53 | 0/53 | 0 | 14/67 | 21 |
| Economic hardship TP1 | 65 | 2/67 | 3 | 2/67 | 3 |
| Economic hardship TP2 | 53 | 0/53 | 0 | 14/67 | 21 |

*Note.* Little’s MCAR: Chi-Square = 233.730, DF = 208, Sig = .107, iterations = 100.

**Supplemental Table 2**

***Participants’ Characteristics and Differences by Attrition***

|  | Participants at both TP1 and TP2  (*n* = 53) | | | |  | Participants only at TP1 (non-participation at TP2) (*n* = 14) | | | |  | Difference | |
| --- | --- | --- | --- | --- | --- | --- | --- | --- | --- | --- | --- | --- |
|  | *n* | % | *M* | *SD* |  | *n* | % | *M* | *SD* |  | *p* |  |
| Year born ^a^ | 52 |  | 41.42 | 12.56 |  | 13 |  | 31.46 | 14.53 |  | .016 | ^a^ |
| Hourly wages ^a^ | 46 |  | 16.74 | 4.24 |  | 11 |  | 14.75 | 4.87 |  | .179 | ^a^ |
| Weekly hours worked ^a^ | 52 |  | 39.10 | 9.55 |  | 12 |  | 33.67 | 9.81 |  | .082 | ^a^ |
| Previous stressors (ACES) ^a^ | 51 |  | 2.82 | 2.76 |  | 10 |  | 2.50 | 3.44 |  | .746 | ^a^ |
| Gender | 51 |  |  |  |  |  |  |  |  |  | 1.00 | ^c^ |
| Female | 48 | 94.1 |  |  |  | 12 | 100 |  |  |  |  |  |
| Male | 3 | 5.9 |  |  |  | 0 |  |  |  |  |  |  |
| Education | 52 |  |  |  |  |  |  |  |  |  | .006 | ^b^ |
| No bachelor’s degree | 22 | 42.3 |  |  |  | 11 | 84.6 |  |  |  |  |  |
| Bachelor’s degree or higher | 30 | 57.7 |  |  |  | 2 | 15.4 |  |  |  |  |  |
| Annual household income | 51 |  |  |  |  |  |  |  |  |  | .185 | ^c^ |
| < $30,000/year | 17 | 33.3 |  |  |  | 7 | 58.3 |  |  |  |  |  |
| >$30,001/year | 34 | 66.7 |  |  |  | 5 | 41.7 |  |  |  |  |  |
| Race | 53 |  |  |  |  |  |  |  |  |  | .34 | ^c^ |
| White | 38 | 28.3 |  |  |  | 8 | 42.9 |  |  |  |  |  |
| Minoritized | 15 | 71.7 |  |  |  | 6 | 57.1 |  |  |  |  |  |

*Note.* ACES = adverse childhood experiences; TP = time point

^a^ = computed with independent samples *t*-test with equal variances assumed. 2-sided *p*-value reported.

^b^ = computed with chi-square test (*df* = 1, 2-sided *p*-value reported)

^b^ = computed with Fisher’s exact test

**Supplemental Table 3**

***Regressions Predicting Physiological and Psychological Stress in Early Childhood Educators (Non-Imputed Data)***

| **Original analysis** |  | | | | | |  |  | | | | | |
| --- | --- | --- | --- | --- | --- | --- | --- | --- | --- | --- | --- | --- | --- |
|  | DV: Change in Physiological Stress (*n* = 23) | | | | | |  | DV: Change in Psychological Stress (*n* = 38) | | | | | |
|  | Unstandardized | |  |  | 95% *CI* | |  | Unstandardized | |  |  | 95% *CI* | |
|  | *b* | *SE* | *t* | *p* | *LB* | *UB* |  | *b* | *SE* | *t* | *p* | *LB* | *UB* |
| Constant | 0.53 | 0.33 | 1.61 | 0.13 | -0.18 | 1.23 |  | 0.45 | 1.24 | 0.36 | 0.72 | -2.08 | 2.97 |
| Wage change | -0.01 | 0.03 | -0.29 | 0.78 | -0.07 | 0.05 |  | -0.09 | 0.11 | -0.78 | 0.44 | -0.31 | 0.14 |
| TP2 household income | -0.22 | 0.21 | -1.05 | 0.31 | -0.67 | 0.23 |  | -1.12 | 0.70 | -1.59 | 0.12 | -2.56 | 0.32 |
| Previous stressors | 0.01 | 0.03 | 0.37 | 0.72 | -0.05 | 0.08 |  | 0.34 | 0.13 | 2.60 | 0.01 | 0.07 | 0.61 |
| Education | 0.22 | 0.21 | 1.06 | 0.31 | -0.22 | 0.66 |  | 0.45 | 0.75 | 0.59 | 0.56 | -1.09 | 1.99 |
| Race White | -0.27 | 0.26 | -1.05 | 0.31 | -0.83 | 0.29 |  | -1.72 | 0.89 | -1.92 | 0.06 | -3.54 | 0.11 |
| Age | 0.01 | 0.01 | 1.05 | 0.31 | -0.01 | 0.02 |  | 0.01 | 0.03 | 0.45 | 0.66 | -0.04 | 0.07 |
| TP2 hormone or steroid use | 0.15 | 0.15 | 0.98 | 0.34 | -0.18 | 0.48 |  |  |  |  |  |  |  |
| TP1 physiological stress | -0.72 | 0.24 | -3.07 | 0.01 | -1.23 | -0.22 |  |  |  |  |  |  |  |
| Stress Composite TP1 |  |  |  |  |  |  |  | -0.44 | 0.12 | -3.79 | <.001 | -0.67 | -0.20 |

*Note.* DV = dependent variable; CI = confidence interval; LB = lower bound; UB = upper bound; TP = time point. Reviewers should be cautioned that the sample size in these analyses is small. As such, we do not interpret these findings, rather only provide this information for reference only.

**Supplemental Table 4**

***Moderating Effects of Wage Change on Changes in Physiological Stress in Early Childhood Educators (Non-imputed Data, n = 23)***

|  | **DV: Change in Physiological Stress** | | | | | |
| --- | --- | --- | --- | --- | --- | --- |
|  | Unstandardized | |  |  | 95% *CI* | |
|  | *b* | *SE* | *t* | *p* | LB | UB |
| (Constant) | 0.64 | 0.36 | 1.77 | 0.10 | -0.14 | 1.41 |
| Wage change x age | 0.00 | 0.00 | 0.79 | 0.44 | 0.00 | 0.00 |
| Wage change | -0.06 | 0.08 | -0.84 | 0.41 | -0.22 | 0.10 |
| TP2 household income | -0.17 | 0.22 | -0.78 | 0.45 | -0.65 | 0.30 |
| Previous stressors | 0.02 | 0.03 | 0.53 | 0.60 | -0.05 | 0.08 |
| Education | 0.24 | 0.21 | 1.14 | 0.28 | -0.21 | 0.69 |
| Race White | -0.28 | 0.26 | -1.07 | 0.30 | -0.85 | 0.29 |
| Age | 0.00 | 0.01 | 0.38 | 0.71 | -0.01 | 0.02 |
| TP2 hormone or steroid use | 0.13 | 0.16 | 0.79 | 0.44 | -0.22 | 0.47 |
| TP1 physiological stress | -0.73 | 0.24 | -3.07 | 0.01 | -1.25 | -0.22 |
| (Constant) | 0.54 | 0.32 | 1.66 | 0.12 | -0.16 | 1.24 |
| Wage change x previous stressors | -0.01 | 0.01 | -1.14 | 0.28 | -0.03 | 0.01 |
| Wage change | 0.03 | 0.04 | 0.67 | 0.52 | -0.06 | 0.11 |
| TP2 household income | -0.21 | 0.21 | -1.01 | 0.33 | -0.66 | 0.24 |
| Previous stressors | 0.02 | 0.03 | 0.73 | 0.48 | -0.05 | 0.09 |
| Education | 0.21 | 0.20 | 1.04 | 0.32 | -0.23 | 0.65 |
| Race White | -0.18 | 0.27 | -0.65 | 0.53 | -0.76 | 0.41 |
| Age | 0.00 | 0.01 | 0.58 | 0.57 | -0.01 | 0.02 |
| TP2 hormone or steroid use | 0.11 | 0.16 | 0.70 | 0.50 | -0.23 | 0.45 |
| TP1 physiological stress | -0.74 | 0.23 | -3.17 | 0.01 | -1.24 | -0.24 |
| (Constant) | 0.57 | 0.35 | 1.63 | 0.13 | -0.19 | 1.32 |
| Wage change x education | -0.03 | 0.06 | -0.47 | 0.64 | -0.16 | 0.10 |
| Wage change | 0.01 | 0.05 | 0.23 | 0.82 | -0.09 | 0.12 |
| TP2 household income | -0.18 | 0.23 | -0.79 | 0.44 | -0.68 | 0.31 |
| Previous stressors | 0.01 | 0.03 | 0.44 | 0.67 | -0.05 | 0.08 |
| Education | 0.24 | 0.22 | 1.12 | 0.28 | -0.23 | 0.71 |
| Race White | -0.28 | 0.27 | -1.03 | 0.32 | -0.86 | 0.30 |
| Age | 0.00 | 0.01 | 0.61 | 0.55 | -0.01 | 0.02 |
| TP2 hormone or steroid use | 0.13 | 0.17 | 0.75 | 0.47 | -0.24 | 0.49 |
| TP1 physiological stress | -0.73 | 0.24 | -3.01 | 0.01 | -1.26 | -0.21 |
| (Constant) | 0.53 | 0.35 | 1.52 | 0.15 | -0.22 | 1.28 |
| Wage change x income | 0.00 | 0.06 | -0.04 | 0.97 | -0.13 | 0.12 |
| Wage change | -0.01 | 0.05 | -0.14 | 0.89 | -0.11 | 0.09 |
| TP2 household income | -0.22 | 0.23 | -0.93 | 0.37 | -0.72 | 0.29 |
| Previous stressors | 0.01 | 0.03 | 0.36 | 0.73 | -0.06 | 0.08 |
| Education | 0.22 | 0.21 | 1.02 | 0.33 | -0.25 | 0.68 |
| Race White | -0.27 | 0.27 | -1.01 | 0.33 | -0.86 | 0.31 |
| Age | 0.01 | 0.01 | 0.88 | 0.39 | -0.01 | 0.02 |
| TP2 hormone or steroid use | 0.15 | 0.17 | 0.89 | 0.39 | -0.21 | 0.51 |
| TP1 physiological stress | -0.72 | 0.25 | -2.95 | 0.01 | -1.25 | -0.19 |
| (Constant) | 0.50 | 0.32 | 1.58 | 0.14 | -0.19 | 1.19 |
| Wage change x race | -0.08 | 0.06 | -1.33 | 0.21 | -0.20 | 0.05 |
| Wage change | 0.03 | 0.04 | 0.83 | 0.42 | -0.05 | 0.12 |
| TP2 household income | -0.22 | 0.20 | -1.07 | 0.31 | -0.66 | 0.22 |
| Previous stressors | 0.02 | 0.03 | 0.69 | 0.51 | -0.04 | 0.09 |
| Education | 0.17 | 0.20 | 0.81 | 0.43 | -0.27 | 0.60 |
| Race White | -0.06 | 0.30 | -0.20 | 0.84 | -0.71 | 0.59 |
| Age | 0.00 | 0.01 | 0.39 | 0.70 | -0.01 | 0.02 |
| TP2 hormone or steroid use | 0.10 | 0.15 | 0.65 | 0.53 | -0.23 | 0.43 |
| TP1 physiological stress | -0.71 | 0.23 | -3.09 | 0.01 | -1.20 | -0.21 |

*Note.* TP = time point; DV = dependent variable; CI = confidence interval; LB = lower bound, UB = upper bound. Reviewers should be cautioned that the sample size in these analyses is small. As such, we do not interpret these findings, rather only provide this information for reference only.

**Supplemental Table 5**

***Moderating Effects of Wage Change on Changes in Psychological Stress in Early Childhood Educators (Non-imputed Data, n = 38)***

|  | **DV: Change in Psychological Stress** | | | | | |
| --- | --- | --- | --- | --- | --- | --- |
|  | Unstandardized | |  |  | 95% *CI* | |
|  | ***b*** | *SE* | *t* | *p* | LB | UB |
| (Constant) | **1.13** | 1.36 | 0.83 | 0.42 | -1.66 | 3.91 |
| Wage change x age | **0.01** | 0.01 | 1.16 | 0.26 | -0.01 | 0.02 |
| Wage change | **-0.48** | 0.35 | -1.35 | 0.19 | -1.20 | 0.25 |
| TP2 household income | **-0.77** | 0.76 | -1.02 | 0.32 | -2.33 | 0.79 |
| Previous stressors | **0.39** | 0.14 | 2.85 | 0.01 | 0.11 | 0.68 |
| Education | **0.48** | 0.75 | 0.64 | 0.53 | -1.05 | 2.02 |
| Race White | **-1.74** | 0.89 | -1.96 | 0.06 | -3.55 | 0.08 |
| Age | **-0.01** | 0.03 | -0.40 | 0.69 | -0.08 | 0.06 |
| Stress composite TP1 | **-0.48** | 0.12 | -3.98 | <.001 | -0.73 | -0.24 |
| (Constant) | **0.48** | 1.27 | 0.37 | 0.71 | -2.12 | 3.07 |
| Wage change x previous stressors | **-0.01** | 0.04 | -0.16 | 0.87 | -0.10 | 0.08 |
| Wage change | **-0.06** | 0.17 | -0.36 | 0.72 | -0.42 | 0.29 |
| TP2 household income | **-1.10** | 0.73 | -1.50 | 0.15 | -2.59 | 0.40 |
| Previous stressors | **0.35** | 0.15 | 2.37 | 0.03 | 0.05 | 0.66 |
| Education | **0.43** | 0.77 | 0.55 | 0.58 | -1.16 | 2.01 |
| Race White | **-1.68** | 0.93 | -1.82 | 0.08 | -3.58 | 0.21 |
| Age | **0.01** | 0.03 | 0.31 | 0.76 | -0.05 | 0.07 |
| Stress composite TP1 | **-0.45** | 0.13 | -3.41 | 0.00 | -0.72 | -0.18 |
| (Constant) | **0.64** | 1.30 | 0.49 | 0.63 | -2.02 | 3.30 |
| Wage change x education | **-0.14** | 0.25 | -0.55 | 0.59 | -0.64 | 0.37 |
| Wage change | **-0.01** | 0.18 | -0.04 | 0.97 | -0.38 | 0.36 |
| TP2 household income | **-1.02** | 0.74 | -1.38 | 0.18 | -2.53 | 0.49 |
| Previous stressors | **0.36** | 0.14 | 2.63 | 0.01 | 0.08 | 0.64 |
| Education | **0.58** | 0.80 | 0.72 | 0.48 | -1.06 | 2.21 |
| Race White | **-1.78** | 0.91 | -1.95 | 0.06 | -3.64 | 0.09 |
| Age | **0.00** | 0.03 | 0.10 | 0.92 | -0.06 | 0.07 |
| Stress composite TP1 | **-0.45** | 0.12 | -3.78 | <.001 | -0.70 | -0.21 |
| (Constant) | **0.43** | 1.30 | 0.33 | 0.75 | -2.24 | 3.09 |
| Wage change x income | **0.01** | 0.25 | 0.05 | 0.96 | -0.49 | 0.52 |
| Wage change | **-0.09** | 0.19 | -0.50 | 0.62 | -0.47 | 0.29 |
| TP2 household income | **-1.14** | 0.81 | -1.40 | 0.17 | -2.81 | 0.52 |
| Previous stressors | **0.34** | 0.14 | 2.52 | 0.02 | 0.06 | 0.62 |
| Education | **0.45** | 0.77 | 0.58 | 0.56 | -1.12 | 2.02 |
| Race White | **-1.71** | 0.91 | -1.88 | 0.07 | -3.57 | 0.15 |
| Age | **0.01** | 0.03 | 0.41 | 0.68 | -0.05 | 0.08 |
| Stress composite TP1 | **-0.44** | 0.12 | -3.60 | 0.00 | -0.68 | -0.19 |
| (Constant) | **0.65** | 1.27 | 0.51 | 0.61 | -1.95 | 3.23 |
| Wage change x race | **-0.21** | 0.26 | -0.83 | 0.41 | -0.74 | 0.31 |
| Wage change | **0.02** | 0.17 | 0.13 | 0.90 | -0.32 | 0.37 |
| TP2 household income | **-0.95** | 0.74 | -1.29 | 0.21 | -2.46 | 0.56 |
| Previous stressors | **0.37** | 0.14 | 2.70 | 0.01 | 0.09 | 0.64 |
| Education | **0.30** | 0.78 | 0.38 | 0.71 | -1.29 | 1.89 |
| Race White | **-1.41** | 0.97 | -1.46 | 0.16 | -3.39 | 0.57 |
| Age | **0.00** | 0.03 | -0.06 | 0.96 | -0.07 | 0.06 |
| Stress composite TP1 | **-0.47** | 0.12 | -3.85 | <.001 | -0.71 | -0.22 |

*Note.* TP = time point; DV = dependent variable; CI = confidence interval; LB = lower bound, UB = upper bound. Reviewers should be cautioned that the sample size in these analyses is small. As such, we do not interpret these findings, rather only provide this information for reference only.

**Supplemental Figure 1**

***Number of Participants in the Study Across Two Time Points***

*Note.* TP = time point; HCC = hair cortisol concentration.
